# Selection of sciatic nerve injury models: implications for pathogenesis and treatment

**DOI:** 10.3389/fneur.2025.1521941

**Published:** 2025-05-07

**Authors:** Pinxi Zhou, Ruhan Zhang, Liangmei Xian, Le Ning, Penghui Lu, Qianyan Liu, Mi Liu

**Affiliations:** School of Acupuncture-Moxibustion, Tuina and Rehabilitation, Hunan University of Chinese Medicine, Changsha, Hunan, China

**Keywords:** sciatic nerve injury, animal models, chronic constriction injury, partial sciatic nerve ligation, sciatic nerve crush injury, sciatic nerve transection, treatments of sciatic nerve sciatic nerve injury

## Abstract

Sciatic nerve injury is one of the most frequent peripheral nerve injuries in the world. The loss of motor and sensory function, along with chronic pain caused by sciatic nerve injury, significantly impacts patients’ quality of life. However, there are numerous restrictions on *in vitro* studies on the regeneration and healing of sciatic nerve damage. In contrast, *in vivo* studies can more accurately mimic clinical pathology through a variety of experimental animal models and a variety of modeling methods. However, the selection of different models has its focus, so this paper reviews the selection of experimental animals, modeling methods, and common treatment protocols. The advantages and disadvantages of each species are discussed, and the modeling methods of five common sciatic nerve injury models, along with their characteristics and applications, are highlighted. Additionally, we briefly summarize the common treatments for sciatica and nerve injury. This review is of great significance for further exploring model selection, the mechanisms underlying sciatic nerve injury, and therapies for nerve regeneration and repair.

## 1 Introduction

**Figure 1:**
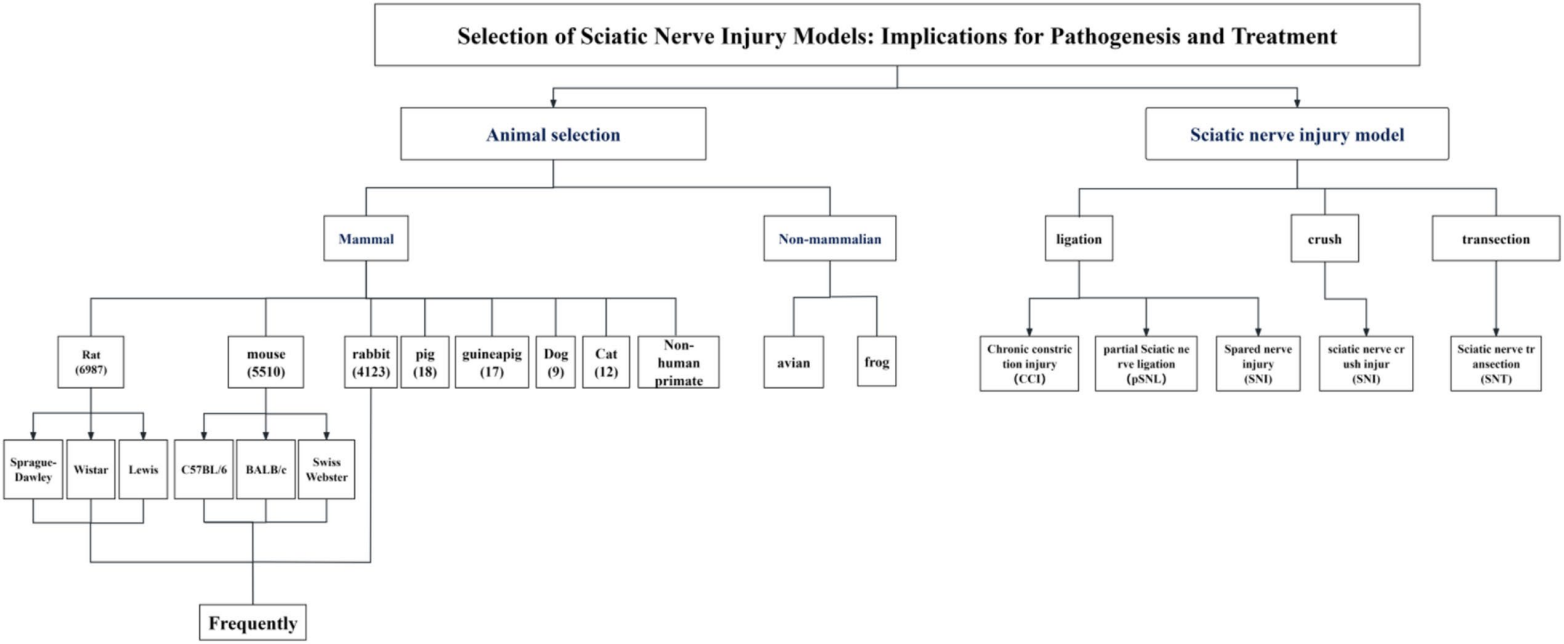
Selection of sciatic nerve injury models: implications for pathogenesis and treatment.

Sciatic nerve injury is one of the most common peripheral nerve injuries and can cause chronic pain as well as a partial loss of motor and sensory function (1). In severe cases, nutritional deficiencies may arise, leading to joint problems, autonomic nervous system malfunction, and paralysis of the regulated muscles, substantially affecting the quality of life of patients and placing an additional burden on their family and community (2–5). Peripheral nerve injury induces plasticity within the spinal cord and cerebral cortex, including sensitization of spinal cord dorsal horn neurons and apoptosis of inhibitory interneurons; these alterations subsequently impact pain signaling and modulation, ultimately resulting in the manifestation of pain (6). Although the nerve has a certain endogenous regeneration ability, the degree of sensory and motor function recovery, the regeneration effect of injured nerve tissue, and the analgesic treatment effect are still not satisfactory after sciatic nerve injury. This clinical problem needs to be solved urgently. Therefore, related basic research has become a research hotspot worldwide (7–9). Currently, a wide variety of mammalian models with varying sizes and anatomical structures, including mice, rats, hamsters, guinea pigs, rabbits, pigs, sheep, cats, dogs, and non-human primates (6), are used in research to assess nerve regeneration and repair function, investigate the mechanism of nerve damage, and examine the effects of various analgesic treatments. The most common peripheral nerve injury models include ligating, crushing, and transecting, with the chronic constriction injury (CCI) model and the partial sciatic nerve ligation (pSNL) model being frequently utilized in investigations of pain mechanisms. The spared nerve injury (SNI) model, the sciatic nerve crush injury (SNI) model, and the sciatic nerve transection (SNT) model are often employed in studies of nerve regeneration and repair. The treatment of peripheral nerve injury mainly involves drugs and surgical repair, including neurotrophic agents, antioxidants, anti-inflammatory drugs, tissue engineering, and stem cell therapy. However, only a few reviews have been conducted on these surgical models and treatments. Therefore, in order to understand the general situation of the above-mentioned research on sciatic nerve injury, this paper focuses on comparing the benefits and drawbacks of each operation-induced sciatic nerve injury model and discusses current typical treatment approaches (Figure 1).

## 2 Search strategy

**Figure 2:**
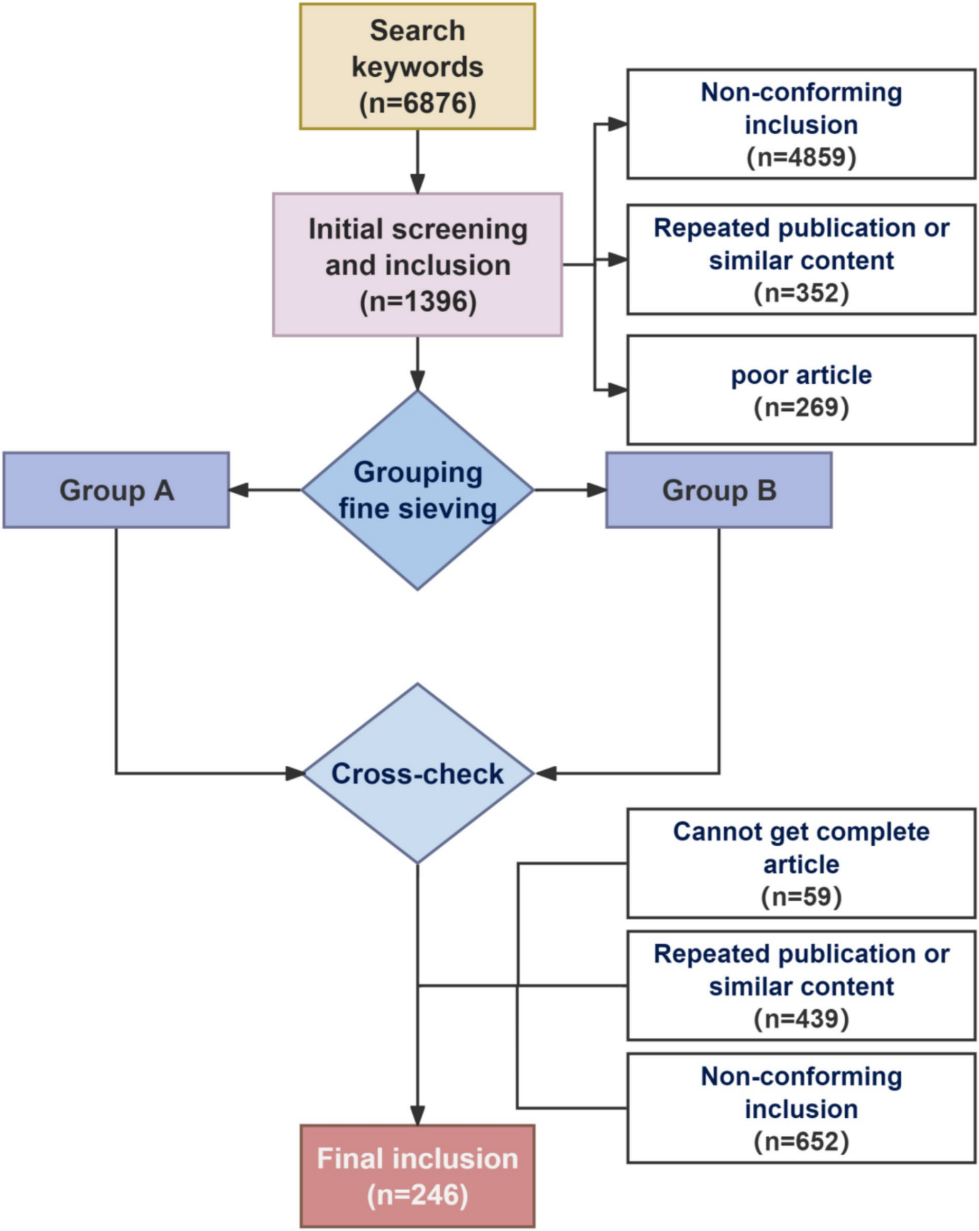
Flowcharts for inclusion and exclusion of literature.

The following keywords were used for searches of publications published from 2014 to 2024 in Chinese and English databases, including China National Knowledge Infrastructure (CNKI), PubMed, and Web of Science (SCIE): “sciatic nerve injury,” “animal models,” “Chronic constriction injury,” “Partial sciatic nerve ligation,” “Spared nerve injury,” “Sciatic nerve crush injury,” “Sciatic nerve transection,” “treatment of sciatic nerve,” “Neurotrophic agents,” “Antioxidant,” “Anti-inflammatory,” “Tissue engineering” and “stem cell therapy.” In addition to focusing on the relevant articles between 2014 and 2024, we also traced many of the original literature on sciatic nerve injury modeling methods to ensure a comprehensive understanding of sciatic nerve injury modeling (Figure 2).

## 3 Animal species

Animal selection for the sciatic nerve injury model is based on a number of design factors and criteria, such as experimental cost, feasibility of operation, ethical considerations, and experimental locations (10). Currently, rats are the most commonly used species, with more than 7,600 entries available in PubMed as of August 2024. With 2,500 entries, mice rank as the second most popular animal species. Rabbits are the third most regularly utilized species, with only approximately 320 entries. In addition, other larger mammals (11) have also been used in experiments, such as pigs (12–14), guinea pigs (15–17), dogs (18–20), and cats (18, 21, 22). However, their utilization rate is low owing to their different evolutionary nerve injury protection and regeneration characteristics. Non-human primates are often used in preclinical testing of nerve scaffolds because of their close similarity to humans (23–25), but strict ethical reviews limit their use. Non-mammalian species, such as avian (26, 27) and frogs (28–30) are widely used in experimental models to study the mechanisms for axon regeneration and nerve repair. While model organisms such as zebrafish and *C. elegans* are widely employed in the investigation of nerve regeneration mechanisms, the absence of a sciatic nerve in these species often leads to their use as simplified models for peripheral nerve regeneration studies (31, 32), or for elucidating the core pathways and cellular biology of *in vivo* axonal regeneration (33, 34).

In terms of animal selection, rats also have many advantages, such as low cost, high genetic homogeneity, and tolerance to surgery (due to their recovery ability), and functional results are easily obtained and identifiable (10). Most importantly, the nerve fibers of rats and humans have similar sizes, bundled tissues, and morphologies (35). Rats can easily repair a reasonable space size using microsurgery and allow for large-sample comparisons. It is often used in experimental studies related to degeneration and repair of peripheral nerve injuries (6, 36). While this model offers utility, it presents notable constraints within the context of nerve grafting investigations. Specifically, the rat sciatic nerve exhibits a capacity for complete regeneration following injury, a stark contrast to the clinically irreversible damage frequently observed in human patients. Furthermore, the critical nerve gap in this model is approximately 1.5 cm, significantly less than the clinical demands, which often necessitate repairs spanning approximately 4 cm in humans. The majority of experimental data is derived from short-gap repairs, typically not exceeding 10 mm. However, clinical repairs necessitate significantly longer gaps, ranging from 5 cm to over 30 cm. This discrepancy in scale renders direct mechanistic analogies problematic, thereby limiting the translational potential of these models (36). For example, in contrast to human nerve injury, the defects produced after nerve rupture injury in rats are fully restored. In addition, the volume, length, and diameter of the sciatic nerve in rats limit the size of devices that can be tested compared to rabbits and other large animals, in turn, reduces the model translational potential (36, 37). While the use of mice in assessing nerve gap repair has limitations in replicating nerve defect size and evaluating the speed and quality of regeneration, they offer advantages in the application of gene manipulation techniques. For instance, the use of transgenic mice enables overexpression or ablation of a particular gene which allows enables researchers to explore the molecular mechanisms of nerve injury and recovery in complex *in vivo* models (38). Coupled with the characteristics of small size, short breeding cycles, and ease of care, mice provide an efficient platform for studying the molecular mechanisms of peripheral nerve repair.

Rabbits are less costly and easier to perform than larger animals. Its in vivo functions, biocompatibility, safety, and clinical relevance are similar to those of humans. The rabbit model can provide both a first-stage test model of artificial neural guidance and an adequate preclinical model (37). Thus, they are often used for neuromorphologic and histological tests. Nevertheless, rabbit studies may also produce a number of adverse side effects (6), such as pressure sores, foot trauma, autotomy, and other unexpected adverse effects. These serious injuries may cause unnecessary pain and suffering to the animals, thus requiring euthanasia and early termination of the experiment for ethical reasons. These complications are incompatible with animal ethics and lower the quality of the nerve repair mechanisms. Large animals, such as cats, dogs, and sheep, are often used to study large neural gaps (39–41). However, unique living and raising circumstances, as well as more intense ethical examination, have significantly decreased their utilization. Porcine and nonhuman primates have nerves closer to those of humans in terms of size, anatomy, and physiology. They are considered the gold standard and are thus used in translational or preclinical investigations (23, 42). However, these models are costly, necessitating state-of-the-art laboratory facilities and expert veterinary care (Table 1).

**Table 1:** Summary table showing the animal selection on SNI.

Animal	Advantages	Disadvantages	Research content
Rats	Low cost, high genetic homogeneity; gentle in character; morphologies; the nerve fibers and bundle tissues similar sizes to human	Species-specific characteristics of neurological regeneration	Degeneration and repair of peripheral nerve injury studies
Mouse	Small size, short reproductive cycle; easy nursing; relatively easy genetic manipulation	Small size of reproducible nerve defects; limited speed and quality of nerve regeneration	Explore the mechanisms of nerve damage and recovery
Rabbits	Biocompatibility, safety, and clinical relevance are close to humans.	Pressure sores, foot trauma, autotomy requiring amputation in severe cases	Research on large nerve damage; Neuromorphological and histological tests
Macro-animal	Large neural gaps	Special living and rearing conditions; stringent ethical scrutiny	Study large neural gaps
Non-human primates	Nerves size, anatomy, and physiology closer to humans	Expensive, highly mature laboratory facilities; pro-fessional veterinary care	Translational or preclinical investigations

## 4 Model methods

### 4.1 Chronic constriction injury (CCI)

**Figure 3:**
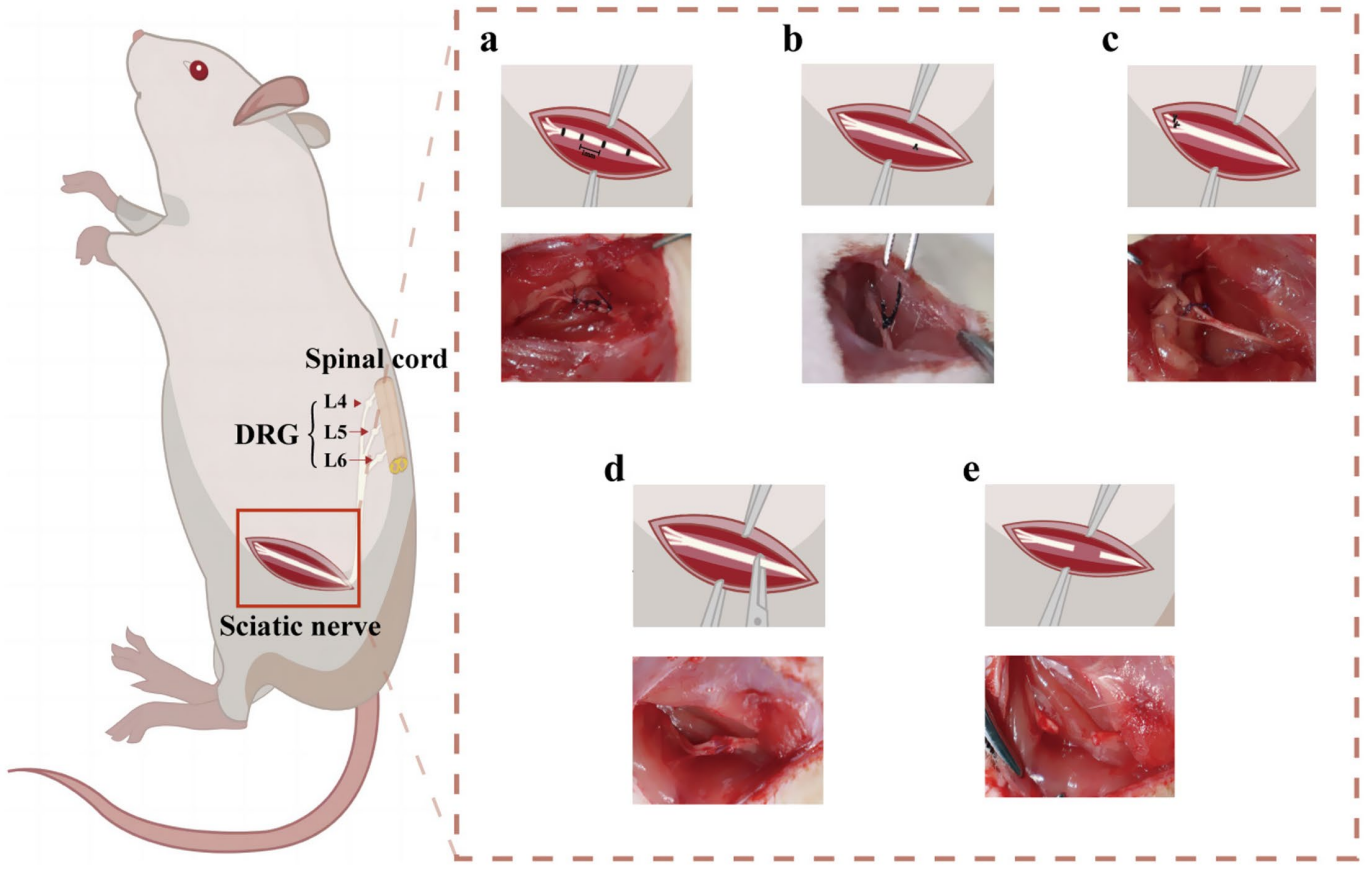
Schematic diagram of SNI model modeling. **(a)** The method used to create CCI in rats. **(b)** The method used to create pSNL in rats. **(c)** The method used to create Spared Nerve Injury in rats. **(d)** The method used to create Sciatic Nerve Crush Injury in rats. **(e)** The method used to create SNT in rats.

The present study adopted internationally recognized methods for the preparation of CCI rat models (43). After the rats were anesthetized, a 1.5 cm incision was made 0.5 cm below the pelvis, and the gluteus maximus and biceps femoris were bluntly dissected to expose the common sciatic nerve. Four loose ligations, each 1 mm apart, were ligated using 4.0 chromium catgut, 2 mm away from the front of the trigeminal branch. The tightness of all ligations was the same, and shaking was observed around the nerves or legs of the rats during ligation. After ligation, the nerve was restored to the original suture and skin layer (Figure 3a).

The CCI model has become a significant tool in neuropathic pain research due to its ease of operation and high experimental reproducibility. The model was initially established using rats as the experimental subject, and was initially established in rats and later expanded to mice (43, 44). Sprague–Dawley (SD) rats and C57BL/6 mice are commonly employed strains in the CCI model. From a pathophysiological perspective, the CCI model exhibits dual mimetic characteristics. On the one hand, it primarily mimics the signs of persistent nerve compression in clinical settings, including trauma or tumor growth (45). On the other hand, it simulates nerve fiber damage mainly located on the surface of peripheral nerves. Following nerve injury, abnormal discharges from damaged nerves trigger changes in the plasticity of the central nervous system, which is thought to be a key mechanism in the generation of pain hypersensitivity, i.e., central sensitization (46). Histopathological analyses have demonstrated that the chronic constriction injury (CCI) model is typified by nerve fiber demyelination, axonal degeneration, and a decrease in Aβ fibers (45, 47), while the majority of C-fibers remain relatively intact, and this specific injury pattern is closely associated with mechanical and thermal nociceptive sensitization in neuropathic pain (46).

As the initial animal model incorporating quantitative assessments of mechanical allodynia (von Frey filament testing) and thermal hyperalgesia (Hargreaves test), the CCI model is extensively utilized in investigations of the peripheral and central nervous systems due to its potential for high similarity to neuropathic pain experienced in clinical settings (48). Compared to partial sciatic nerve ligation (pSNL), the CCI model demonstrates more pronounced effects in inducing mechanical allodynia, thermal hyperalgesia, and cold allodynia (4, 49, 50), and reliably elicits spontaneous pain-like behaviors (51, 52), along with fewer ectopic pain-like behaviors (53), typically within 10–14 days post-surgery. Thus, this approach is commonly used in behavioral studies. However, in this model, in addition to causing neuropathic pain, the chromium line also leads to inflammation (54–56), and the different tensions of the ligation can significantly affect the number and type of damaged fibers (57) (Table 2).

**Table 2:** Summary table showing the model methods on SNI.

Models	Characteristic	Disadvantages	Research content
CCI	Simple and high reproducibility; mechanical pain; thermal pain; cold hyperalgesia, spontaneous pain-like behaviors	Inflammation; influence of different tension	Chronic nerve compression; assess neuropathic pain; behavioral studies
pSNL	Rapid onset; tactile painless; hyperalgesia and induced to the contralateral side	Degenerated other fibers; injured and uninjured primary afferent nerves are mixed; difficult to study the changes of the DRG	Nerve contusion; assess neuropathic pain
SNI	High reproducibility; mechanical pain	Thermal threshold hardly changes	Biochemical and cellular studies of nerve and skin areas; retrograde tracing; specific nerve therapy; behavioral evaluation
SNI (crush)	Simple and high reproducibility; commonly for preclinical studies	The pressure is uncontrollable	The therapeutic strategies and biology of peripheral nerve regeneration
SNT	The most serious nerve damage; motor incoordination; mechanical hyperalgesia; abnormal tactile pain; spinal cord pain; axonal degeneration	Easy form Neuroma and further cause pain or sensory abnormality, excessive self-resection	Simulate phantom limb pain, tissue engineering materials on regeneration and repair after nerve injury

### 4.2 Partial sciatic nerve ligation (pSNL)

After anesthesia, the upper segment of the sciatic nerve was treated with 8–0 silk suture, and 1/3–1/2 its diameter was ligated. The strength of ligation was measured using the diameter of the nerve with little contraction. Following ligation, the nerve was restored to the original suture and skin layer (58) (Figure 3b).

The pSNL is a model of pathogenic-type diversity syndrome induced by partial nerve injury and is maintained by sympathetic nerve activity. The model was initially developed in rats and later generalized to C57BL/6J mice (59) and is still mainly used in SD rats or C57BL/6J mice, and the intensity of mechanical nociceptive sensitization varies between strains, with C57BL/6J mice showing strong and persistent mechanical nociceptive sensitization, and C3H/HeSlc mice being less sensitive than other mice Strain (60). It embodies many characteristics of human pain, including the rapid onset of tactile painless hyperalgesia, mirror images, and dependence on sympathetic output (61). The partial injury caused by this model produces selective amplification of the response. Hyperalgesia, mainly heat pain and mechanical ectopic pain, develops within a few hours after surgery and lasts for several months (45). Spontaneous pain and signs such as considerable exercise hypersensitivity and hyperalgesia can be induced on the contralateral side (58, 62). However, other fibers in the unligated part of the nerve in this model were degraded due to the destruction of the nerve bundle membrane and the local vascular system. Injured and uninjured primary afferent nerves are mixed; hence, researching the alterations in the dorsal root ganglion is challenging (45) (Table 2).

### 4.3 Spared nerve injury (SNI)

After anesthesia, on the lateral aspect of the left thigh, the proximal and distal portions of the biceps femoris were cut, exposing the sciatic nerve and its three distal branches (from left to right: common peroneal nerve, tibial nerve, and sural nerve). The distal 2–3 mm common peroneal nerve and tibial nerve were tightly ligated, and then sectioned with micro-scissors approximately 5 mm away from the ligation. During surgery, extra attention must be used throughout the surgery to prevent contacting or straining the sural nerve that is still intact (63) (Figure 3c).

The SNI model was originally established for SD rats and can be applied to both rats and mice, with C57BL/6 mice also being a commonly used strain in newer studies (64). The model has high reproducibility and success rates (65). Changes in nearby intact sensory neurons and wounded main sensory neurons may be directly studied, allowing for an analysis of their respective roles in the pathophysiology of pain. Owing to the presence of the sural nerve, it is capable of rapid allergy in skin areas near denervation, with considerable and long-term changes in mechanical sensitivity and heat reactivity (65). These alterations bear many similarities to clinical neuropathic pain characteristics, including mechanical pain, which is longer than CCI (66), and demonstrated a sustained rise in the ipsilateral response to suprathreshold thermal and cold stimulation (65). The SNI model had a clear anatomical distribution: at the distal end of the injury, the injured and non-injured nerves were not intermingled (67). The mixture was limited to the boundary region between the damaged tibial nerve and the intact sural nerve model. Therefore, injured and uninjured sites are easily identified and modified, allowing biochemical and cellular studies of both damaged and undamaged nerves and skin areas at the peripheral (neural tissue, dorsal root ganglia) or central (spinal dorsal horn) levels, further retrograde tracing, specific nerve therapy, and behavioral evaluation (68) (Table 2).

### 4.4 Sciatic nerve crush injury (SNI)

A single oblique incision was made at the lower edge of the hip joint of the hind limb, and the lateral muscle was separated by ophthalmic shear force until the sciatic nerve was exposed. A mark was made on the sciatic nerve membrane using an 8–0 suture needle at a fixed position near the piriform fossa in the middle of the rat femur. Professional pinch forceps were used to pinch the distal nerve near the marker with a 3-mm length for 30 s (10 s each) before muscle and skin suture (69). The same individual performed all surgical preparations to minimize differences among the animals (Figure 3d).

In the rat model of sciatic nerve crush injury, microscopic surgery is not required, which greatly reduces the experimenter’s technical requirements. In this model, functional changes are mainly caused, leading to permanent anatomical damage (70, 71). It is similar to ligation, but without destroying the nerve connective tissue, especially the epineurium (72), it breaks only the continuity of axons and, therefore, does not induce losses in the continuity of the nerve trunk (73, 74). Thus, the proximal and distal nerve segments of the lesion site remain connected, enabling the severed axons to recover and return to the original nerve target via the best regeneration pathway—the distal Wallerian degeneration regeneration environment (61). These characteristics make the sciatic nerve crush injury model more appropriate for studying the biology of peripheral nerve regeneration and developing therapeutic strategies to enhance nerve regeneration (75). The model can be evaluated by behavioral tests for recovery of sensory and motor functions. Mechanical abnormalities of pain were quantitatively analyzed using the von Frey test to determine the Paw withdrawal threshold (PWT) or in combination with the pinprick assay (76). Recovery of thermal hyperalgesia could be detected by the Hargreaves test for Paw withdrawal latency (PWL) (The gut metabolite indole-3 propionate promotes nerve regeneration and repair). Recovery of motor function can be systematically evaluated using a grip strength test, Sciatic Function Index (SFI), and toe extension (8). For histopathological verification, SCG10 immunofluorescence staining assessed axonal regeneration (77), while PGP9.5 staining specifically traced the degree of reinnervation of cutaneous sensory nerve endings (78). The high reproducibility of the model makes it easier to identify changes in whole tissue at the cellular and molecular levels. Hence, it is particularly suitable for research on the changes over the time course of regeneration. Additionally, alterations in the results of nerve regeneration after sciatic nerve crush injury can serve as preclinical indicators of the efficacy of therapeutic agents or tissue-engineering strategies for nerve regeneration (Table 2).

### 4.5 Sciatic nerve transection (SNT)

After anesthesia, the proximal and distal portions of the biceps muscle were severed on the lateral surface of the thigh. After sciatic nerve exposure, the nerve was transected approximately 5 mm to prevent nerve reconnection due to regeneration (79). The muscle and skin layers were closed after surgery. In order to prevent axon regeneration, which is more prominent in animals than in humans and happens spontaneously following full transection, even in the absence of nerve repair, extra care should be taken to flip the proximal nerve stump and suture it to nearby tissue. However, clinically, if there is no material loss, the broken end anastomosis can restore nerve continuity, that is, the direct suture of the two-nerve stump. If the broken end is defective, nerve transplantation can be performed, allowing the axon to regenerate the original motor and sensory targets along the distal end (80) (Figure 3e).

The sciatic nerve transection model is an extremely ancient animal neuropathic pain model with the most serious nerve damage (81). It is a suitable model for phantom limb pain, mimicking the human experience of pain anesthesia after amputation, where pain is felt without any sensory input. This model also causes damage to the adjacent cryptic nerve (82), resulting in the complete denervation of the distal hind limb, mechanical hyperalgesia, abnormal tactile pain, increased spinal cord pain, motor incoordination, and axonal degeneration. In addition to causing motor function impairment, it can also cause autonomic dysfunction (83). After transfection, the proximal nerve loses its corresponding distal nerve and innervation target, easily grows in all directions, and is entangled with the surrounding hyperplastic fibers, in the local formation of scar-like structures, namely neuroma. Neuroma can further cause pain or sensory abnormality through tearing and stretching of surrounding tissues (84). In this model, self-resection of animals occurs (self-attack and cutting of denervated limbs in injured animals), which is considered to be accompanied by neuropathic pain and is ethically questioned because of the possibility of excessive self-resection (Table 2).

## 5 Treatment options for sciatic nerve injury

### 5.1 Neurotrophic agents

**Figure 4:**
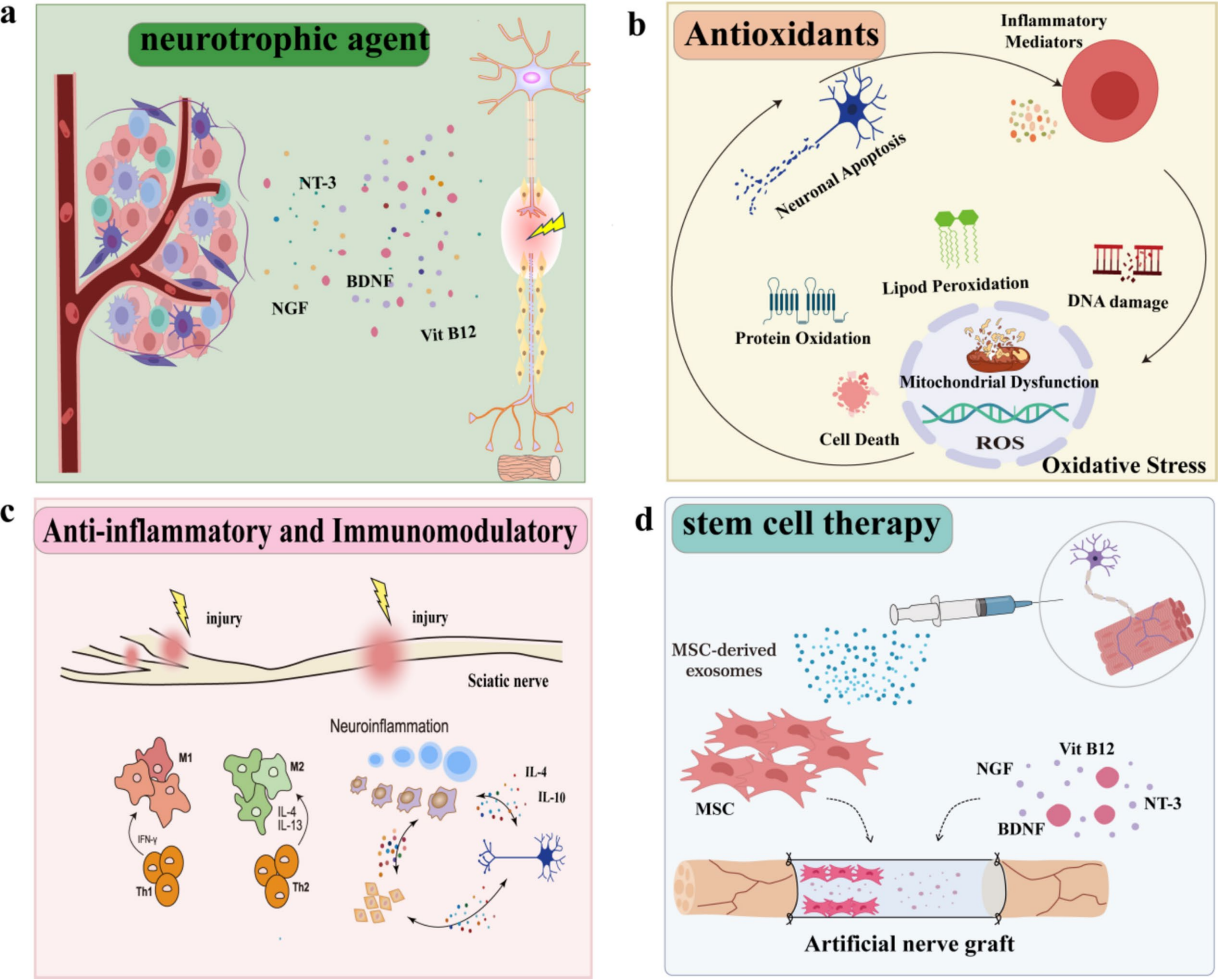
Treatments of sciatic nerve injury. **(a)** Neurotrophic agent. **(b)** Antioxidants. **(c)** Anti-inflammatory and immunomodulatory. **(d)** Tissue engineering and stem cell therapy.

Neurotrophic agent can aid the regeneration and repair of injured nerve by reducing cell death, participating in nerve metabolism and improving blood circulation. It mainly includes neurotrophic factors (e.g., NGF, BDNF), B vitamins and their derivatives (e.g., B1, B12, mecobalamine) (Figure 4a and Table 3).

**Table 3:** Common treatments options for sciatic nerve injury.

Treatments	Common drugs or materials
Neurotrophic agents	NGF, BDNF; Vit B1, Vit B12, mecobalamine
Antioxidant	Vit E, Vit C, glutathione, pyrroloquinoline quinone, melatonin
Anti-inflammatory	ibuprofen, meloxicam, indomethacin, diclofenac, acetaminophen; dexamethasone
Tissue engineering and stem cell therapy	PLA, PGA, PLGA, PCL; collagen, chitosan, silk fibrillin; gelatin, hyaluronic acid, fibrin

Accurately, in peripheral nerve injury, the increase or enhancement of neurotrophin activity can protect the damaged neurons and promote neuronal axon regeneration and nerve repair, which provides a potential pathway for molecular therapy to enhance nerve regeneration (85). Numerous lines of evidence suggest that NGF is essential for the healing of nerve damage (86, 87), promoting the proliferation and differentiation of neurons, enhancing the ability of axon regeneration, regulating the recovery of damaged nerve structure and function, and has the potential to promote the regeneration of peripheral nerves (88). Nevertheless, as the unnecessary side effects of intravenous NGF on the peripheral nerve are limited by its low stability, short half-life, and high cost (89, 90), it is not encouraged to try exogenous NGF in humans (91). In this light, using safe strategies to induce increased NGF production may be an optimal choice. Let-7 is one of the most conserved members of the MicroRNA (miRNAs) family, involved in regulating the fate of neurons and influencing their degeneration and regeneration (92). Evidence shows that regulating the let-7/NGF axis can reduce oxidative stress, thereby promoting myelination, nerve regeneration, and the restoration of nerve function after peripheral nerve injury (93, 94). In addition, collagen-bound NGF can be localized at the site of nerve injury in the rat model of sciatic nerve crush injury, facilitating nerve repair and enhancing functional recovery (69). Additionally, Sang et al. found that curcumin can stimulate the release of NGF, thereby playing a protective role in damaged neurons and further activating TrkA and PI3K/Akt cell survival signals (95). Brain-derived neurotrophic factor (BDNF) is a neuropeptide and involved in many aspects of synaptic formation and plasticity (96–99). Certainly, previous research has shown that viral vector-mediated BDNF gene therapy has confirmed the protective effect of BDNF in neuronal survival after injury (100). Likewise, according to Catia Lopes et al., injecting non-viral TMCSH-HC nanoparticles intramuscularly with therapeutic transgenes encoding neurotrophic factors may be able to prevent long-term neurodegenerative changes and thus accelerate nerve regeneration (101).

The vitamin B (Vit B) complex, in particular vitamin B12, has neuroprotective properties as well as anti-apoptotic and anti-necrosis effects on neurons (102). Vit B complex or Vit B12 may reduce the expression of pro-inflammatory cytokines and increase the expression of anti-inflammatory cytokines in the process of peripheral nerve injury or inflammation, which is propitious to the regression of nerve inflammation (103). Moreover, it can also promote the regeneration of myelinated nerve fibers and the proliferation of Schwann cells by boosting the quantity of Schwann cells, myelinated nerve fibers, and the diameter of axons (104, 105). Noticeably, Several studies have reported that Vit B12 can improve nerve conduction, promote the regeneration of injured nerves, and inhibit the spontaneous ectopic discharge of injured primary sensory neurons (106). It can promote regeneration and functional recovery of injured sciatic nerves by upregulating BDNF expression (89). It has also been proposed that tissue levels of Vit B complex and Vit B12 change as compression-induced peripheral nerve injury progresses and may help to speed up neuron regeneration (107).

Moreover, in addition to promoting neuroregeneration and repair, multiple studies have indicated that Vit B12 can also be employed in pain management, alleviating neuropathic pain through various mechanisms, including the promotion of myelination, enhancement of nerve regeneration, and reduction of ectopic discharges (108–110). In summary, B vitamins exhibit therapeutic potential in the context of neuroinflammation and neural regeneration, concurrently ameliorating pain symptomatology in affected patients.

### 5.2 Antioxidant

Antioxidants exhibit a dual role in the neuroprotective effects: they can promote neuroregeneration and indirectly achieve analgesic effects by modulating pain signaling pathways. In PNI, inflammation leads to a buildup of oxidizing agents and free radicals (111). Notably, Oxidative stress plays an important role after nerve injury, ROS can induce neuronal apoptosis via the mitochondrial pathway (112) and regulate the expression of some apoptotic genes involved in pain generation (113) and central sensitization of neuropathic pain (114, 115). Multiple lines of evidence suggest that pro-oxidant enzymes or ROS scavengers can reduce injury hypersensitivity in rodent models of neuropathic pain (116–118). Hence, the use of antioxidants after peripheral nerve injury can reduce free radical production and protect nerve cells from oxidative damage (119). Commonly used antioxidants include Vitamin E, Vitamin C, glutathione, pyrroloquinoline quinone, and melatonin (Figure 4b and Table 3).

Vitamin C (Vit C) and Vitamin E (Vit C) are potent antioxidants (120–122). Vitamin E deficiency affects the central and peripheral nervous system and may lead to peripheral neuropathy (123). Morani et al. showed that vitamin E inhibits neuropathic pain after sciatic nerve constriction in rats (124). According to certain studies, the underlying process via which vitamin E restores sciatic nerve function following crush injuries may include the regulation of oxidative stress pathways (125, 126). Research indicates that administering exogenous Vit C and Vit C in combination has a synergistic analgesic effect. It also prevents neuropathic pain behavior following peripheral nerve damage and the early behavioral reaction to formalin injections (117). Further, the combination of pregabalin and vitamins significantly reversed sciatic nerve Wallerian degeneration and inflammatory response (127). Similarly, in another study, researchers proved that a combination of Vit C and gabapentin bacillus for nerve injury increases anti-inflammatory and anti-injury effects (128, 129).

Interestingly, certain antioxidants facilitate structural repair and functional recovery by enhancing the expression of regeneration-associated proteins. Pyrroloquinoline quinone (PQQ), an anti-lipid peroxidation antioxidant, enhances NGF synthesis in Schwann cells, improving the remarkable ability of peripheral nerve regeneration after injury (130). Interestingly, a study in SNT rats revealed that Vit E combined with PQQ treatment accelerates and improves peripheral nerve regeneration (131). In addition, melatonin has a stronger antioxidant capacity (132), and a study has demonstrated that melatonin nerve-directed conduits improve the immunological milieu by minimizing oxidative stress, inflammation, and mitochondrial dysfunction, providing energy to the nerves, and reducing neuronal apoptosis, which promotes neural debris removal and neural proliferation (111). There is accumulating evidence that antioxidants such as curcumin, isoquercitrin, and ginger oil, and mitochondrially targeted antioxidant such as MitoTEMPO have crucial roles in axonal regeneration after peripheral nerve injury and in reducing pain hypersensitivity (133–136). Furthermore, the mitochondrially-targeted small molecule M1 offers similar ability to promote axon regeneration following sciatic nerve injury, similar to MitoTempo (137).

### 5.3 Anti-inflammatory drugs

Inflammation is an inevitable process in the process of sciatic nerve injury and repair. Inhibition of inflammation can reduce the degree of nerve injury and neuropathic pain and promote nerve repair and regeneration (138, 139), thereby improving pain. Therefore, neuroinflammation and neuropathic pain can be partially reversed by inhibiting essential pro-inflammatory cytokines and chemokines. Anti-inflammatory drugs such as non-steroidal anti-inflammatory drugs (e.g., ibuprofen, meloxicam, indomethacin, diclofenac, acetaminophen) and glucocorticoids (e.g., dexamethasone) can regulate inflammation-related signaling pathways, such as inhibiting the activation of NF-κB and regulating the expression of cytokines (e.g., TNF-*α*, IL-1β), reduce nerve inflammation and local tissue damage, promote nerve regeneration, and widely used in the treatment of nerve injury (140) (Figure 4c and Table 3).

The crucial of NF-κB in the repair of peripheral nerve injury has been recognized for several years, which may control the release of pro-inflammatory cytokines and express the main proteins in cytokine-induced inflammatory and immune responses (141). The activation of the NF-κB signaling pathway has been shown to have a vital effect on the survival and plasticity of neurons (142, 143). In addition, in a CCI rat study, it was revealed that local inhibition of NF-κB activity in dorsal spinal cord glial cells was sufficient to reduce thermal hyperalgesia and mechanical ectopic pain in CCI rats and may be related to the prevention of IL-6 and iNOS expression (144). Apparently, diclofenac sodium is a common non-steroidal anti-inflammatory medication which has analgesic, anti-inflammatory, and antipyretic properties (145, 146). The process could include preventing NF-κB from being activated, which would impact the synthesis of pro-inflammatory cytokines like IL-6 (147). In addition, it has been suggested that diclofenac sodium (DS) improves the mechanical extraction threshold and cold ectopic pain induced by SNI (148). Indeed, acetaminophen is a multimodal analgesic. A study identified that acetaminophen can effectively reduce TNF-αand IL-βin rats induced by CCI and exert anti-inflammatory and analgesic effects (149). This study further verified that the combination of L-carnosine and acetaminophen increased antioxidant capabilities and eliminated ROS by reducing GSH and stimulating the synthesis of antioxidant enzymes (SOD). An interesting animal study used celecoxib loaded with near-infrared labeled nano-emulsion (NE) to act on CCI-induced rats. It was found that CXB-NE, in turn, affected the regulation and expression of genes in sciatic nerve induced by CCI through targeted inhibiting macrophage COX2 and lowering macrophage PGE2, which was characterized by decreased expression of several neuroinflammatory genes (150, 151). Similarly, dexamethasone is an effective anti-inflammatory glucocorticoid. Dexamethasone’s immunosuppressive action and neurotrophic potential can improve the functional and morphological indexes of the injured peripheral nerve by reducing the infiltration of inflammatory cells (102), contributing to the production of inflammatory mediators, lessening the severity of Waller’s degeneration, and delaying the clearance of myelin fragments after PNI (152, 153), improving the functional and morphological indexes of the injured peripheral nerve. Actually, it can up-regulate BDNF and increase the immunoreactivity, fibrosis, and oxidative stress of NGF in nerve tissue (154, 155).

The above studies revealed the potential of traditional drugs to alleviate neuroinflammation by targeting the regulation of NF-κB and other pathways. In addition, some natural products have gradually become the new focus of nerve repair research due to their multi-target synergistic mechanism of action. Berberine (BBR) is a natural isoquinoline alkaloid which has the effects of anti-inflammation, antioxidation, and anti-apoptosis (156). BBR has been shown to support axon regeneration, neurite outgrowth, and remyelination following damage in the PNS (157). Additionally, BBR significantly reduced the pro-inflammatory M1 polarization of macrophages by inactivating NLRP3 inflammatory bodies, confirming its anti-inflammatory and neuroprotective benefits in PNI (158). Of note is that Tian et al. summarized the neuroprotective properties of BBR. They found that BBR mediates neuroprotection by inhibiting MAPKs, AMPK, NF-κB, TLR4, and NLRP3 pathways and directly regulating the secretion of inflammatory factors (159).

### 5.4 Tissue engineering and stem cell therapy

In addition to drug therapy, tissue engineering and stem cell therapy are also hot topics of current research. Through the implantation of specific cells, biological scaffolds or stem cells cultured *in vitro*, nerve regeneration and repair can be promoted and better therapeutic effects can be provided (Figure 4d and Table 3).

This treatment scheme is mostly used in the sciatic nerve transection model, usually using graft or biomaterial engineering structure, namely synthetic nerve guide conduit (NGCs) (160, 161). Grafts can be divided into Autografts and Allografts (162). The autograft is derived from a healthy part of the patient’s body and is most commonly used in blood vessels, muscles, and tendons, thus reducing the chance of immune rejection (163). However, tissue damage at the donor site causes morbidity at the donor site, which limits its applicability. In allografts, the tissue is taken from a different member of the same species donor. Although this technique is characterized by increased tissue availability, it carries a small risk of disease transmission and immune response. Owing to the limitations of these two methods and advances in tissue engineering in the field of nerve transplantation, nerve catheters made of different materials have also been increasingly employed. These catheters are biocompatible synthetic polymers and natural polymers. The most commonly used materials for manufacturing synthetic polymers include polylactic acid (PLA), polyglycolic acid (PGA), polylactic acid, polyglycolic acid copolymer (PLGA), and polyhexide lactone (PCL) (164–167). Natural polymers include collagen, chitosan, and silk fibrillin (163, 168, 169). Other materials used to observe the effects of different tissue engineering materials on regenerative repair after nerve injury include gelatin (170, 171), hyaluronic acid (172), and fibrin (173).

In addition, the implantation of biosynthetic nerve conduits containing neuroprotective cytokines is also an effective treatment for the repair and regeneration of sciatic nerve injuries. A recent study (174) selected Vit B12 and NGF as anti-inflammatory and regenerative drugs. It developed a response cascade drug delivery stent (RCDDS) which can regulate the kinetics of drug release by opening the polymer chain in layers triggered by ultrasound. The results showed that the controllable release of Vit B12 reduced the degree of inflammation at the site of PNI by diminishing the local ROS levels and causing macrophages to transform into anti-inflammatory M2 cells. Clearly, Mesenchymal stem cells (MSCs) are adult-derived stem cells which can secrete various nerve growth factors and stimulate peripheral nerve regeneration (175, 176). It should also be noted that combined therapy (stent + stem cell + drug) is considered to be the best strategy for repairing nerve defects (154). Studies have shown that the combination of MSCs and melatonin can effectively promote the repair and regeneration of sciatic nerve and improve the function of damaged meridian tissue by reducing lipid peroxidation and increasing the level of BDNF (177). In addition, Wei Zhang et al. (178) created a novel method of treating sciatic nerve damage by sandwiching MSCs laden with EPO with chitosan nerve conduit (EPO/Chi), which may greatly speed up nerve healing and enhance morphological recovery. Interestingly, in another study (179), a biodegradable conductive hydrogel was created by combining polydopamine-modified silicon phosphorus (SiP@PDA) nanosheets with a combination of methacryloyl gelatin and acellular extracellular matrix (GelMA/ECM). MSCs cultured in 3D in GelMA/ECM-SiP@PDA conductive hydrogel showed a significant up-regulated in the expression of genes related to the differentiation of SC-like cells, promoting myelin formation and axonal regeneration.

## 6 Conclusion and future perspectives

In this paper, the model and treatment of sciatic nerve injury were reviewed, the results show that rats, mice, and rabbits are the most commonly used animals for the development of such models, although animals of different species and sizes can be used for sciatic nerve injury modeling. The primary considerations for this choice are the economic cost, nursing difficulty, experimental operability, and ethical welfare issues (10). The shape and size of nerve fibers and the distribution of nerve stem in rats are similar to those in humans (35), and they have many advantages, such as resistance to infection, vitality, and low cost. Therefore, rats are often selected for physiological function testing. Mice are easy to reproduce and convenient for genetic manipulation and are often used in the study of drugs to repair sciatic nerve injury. Compared to the sciatic nerve of rats and mice, the sciatic nerve of rabbits is slightly closer to the human sciatic nerve morphology, and it is easy to observe and study the nerve microstructure. Therefore, it is often used in the morphological and histological detection of the sciatic nerve (37). We also discovered that sciatic nerve research in bigger mammals and non-mammalian models has been declining, and the relevant literature is rather old. As a result, the study of the vast nerve space requires more investigation.

Currently, basic research on sciatic nerve injury is mainly aimed at finding effective analgesic treatments and exploring the mechanisms of nerve repair and regeneration. In terms of model replication, the main differences focus on the location and nature of the injury. The sites of the injury include distal or proximal nerve roots and spinal cord roots, and the nature of injury is characterized by tight or loose nerve ligation, compression, transection, and neuroinflammation (74, 81). Ligation is used as a modeling method to study the changes in the proximal and distal nerves, other centers, including neuronal cell bodies, and distal anatomical structures, including muscles. It can induce functional changes resulting in spontaneous pain-like behaviors, mechanical hyperalgesia, thermal hyperalgesia, and cold hyperalgesia (180, 181). Therefore, it is commonly used to explore rational chronic pain in sciatic neuropathy, its analgesic mechanism, and inflammatory factors. However, the most common problem with the ligation model is that the surgical technique, proficiency of the experimenter, and ligation strength are difficult to control consistently. These factors are key to the success of model replication and have a particular impact on the effect of the model. In the crush injury model, nerve edema is caused by nerve injury. The continuity of the nerve trunk is preserved by interrupting the continuity of most axons without damaging nerve connective tissue (9, 73, 74), including the epineural membrane. It induces the regeneration of injured axons along the best regeneration pathway (61, 80). This model is more suitable for studying the architecture and activity of nerves in order to investigate the recovery of motor and sensory functions after nerve injury. In contrast, the nerve transection model is help in studying the distal nerve trunk and skeletal muscle denervation to investigate effective preventive strategies. This model involves irreversible disconnection of neurons from their respective distal nerve destinations (79, 81), resulting in significant loosening of the sensory or motor function and muscle atrophy.

In the choice of treatment, the current drug therapy is mainly neurotrophic agents, antioxidants and anti-inflammatory drugs, and surgery is combined with tissue engineering and stem cell transplantation. Although a large number of studies have proved the effectiveness of drug therapy, the half-life and side effects of drugs limit the development prospect of drug therapy. Therefore, the combined treatment of tissue engineering scaffolds, stem cells and drugs is considered to be the best strategy for repairing nerve defects (154). However, only 5 physical damage models of the sciatic nerve were reviewed in this study, and freezing injury and pharmacological (chemical) injury were excluded, which may not be sufficient to offer a thorough overview of current research on sciatic nerve injury models. Furthermore, in the treatment of sciatic and peripheral nerves, only medication therapy, tissue engineering, and stem cell therapy are briefly covered, while developing technologies such as electrical stimulation, magnetic therapy, laser therapy, and rehabilitation therapy are not included. These will be the subject of further investigation.

Overall, an appropriate animal model of sciatic nerve injury can provide valuable information for the pathogenesis and clinical treatment of sciatic nerve injury and is also an indispensable part of understanding the pathophysiological mechanism of sciatic nerve injury and developing new drugs. The advantages and disadvantages of various models and animals in the preparation of the sciatic nerve model are well understood. The model should be designed based on the specific research purpose of the experiment, with selection of the most appropriate animals. Such a model would provide a reliable basis for subsequent studies on the repair and regeneration mechanisms after sciatic nerve injury. Our study provides an overview of species selection commonly used in sciatic nerve injury models but does not address potential differences within species, particularly at the strain level. This limitation is noteworthy given that differences in genetic background between animal strains may affect nerve regeneration outcomes. With continuous advances in science and technology, more effective sciatic nerve injury models must be developed. New methods for sciatic nerve injury modeling should be introduced, which will provide a valuable clinical and scientific basis for revealing the pathogenesis of sciatic nerve injury and contribute to the development of new drugs and other treatments.
